# High Detection Rate of Rotavirus Infection Among Children Admitted with Acute Gastroenteritis to Six Public Hospitals in Luanda Province After the Introduction of Rotarix^®^ Vaccine: A Cross-Sectional Study

**DOI:** 10.3390/v16121949

**Published:** 2024-12-20

**Authors:** Dikudila Vita, Manuel Lemos, Zoraima Neto, Mathebula Evans, Ngiambudulu M. Francisco, Filomeno Fortes, Ema Fernandes, Celso Cunha, Claudia Istrate

**Affiliations:** 1 Faculty of Medicine, Agostinho Neto University, Luanda P.O. Box 116, Angolamanuel.lemos90@yahoo.com.br (M.L.); emacfernandes@hotmail.com (E.F.); 2 National Institute for Health Research, Luanda P.O. Box 3635, Angola; 3 School of Health Systems and Public Health, Faculty of Health Science, University of Pretoria, Pretoria 0084, South Africa; evans.mathebula@abbott.com; 4 Global Health and Tropical Medicine (GHTM), Institute of Hygiene and Tropical Medicine (IHMT), NOVA University (UNL), 1349-008 Lisbon, Portugal; filomenofortes@gmail.com (F.F.); ccunha@ihmt.unl.pt (C.C.); 5 Associate Laboratory for Animal and Veterinary Sciences (AL4AnimalS), Interdisciplinary Center for Research in Animal Health (CIISA), Faculty of Veterinary Medicine, University of Lisbon, 1300-477 Lisbon, Portugal

**Keywords:** rotavirus, acute gastroenteritis, children, vaccine, Luanda, Angola

## Abstract

Rotavirus group A (RVA) is a major cause of pediatric acute gastroenteritis (AGE). Vaccination is an effective public health strategy and Angola implemented it in 2014. This hospital-based study aimed to estimate the prevalence of RVA infection and the severity of AGE in children under five years of age treated at six hospitals in Luanda Province. Between April 2021 and May 2022, 1251 fecal samples were screened by an immunochromatographic rapid test (SD Bioline). Data on socio-demographic profile, nutritional status, and clinical assessment were obtained. The association of RVA infection and AGE severity with possible risk factors was evaluated with a binary logistic regression model. Overall, the detection rate was 57.8% and girls tend to be more often infected than boys (55.2%). Infection was more common in the youngest group (1 to 6 months, 60.3%). Important sources of RVA infection were drinking water kept in tanks (57.9%) and private sanitary facilities with piped water (61%). Surprisingly, according to the Vesikari Scale score, the most severe symptoms were observed in children vaccinated with two doses (80.7%). RVA prevalence remains high despite vaccination, and further studies should address the association between infection sources and disease severity, as well as the causes underlying vaccine (un)effectiveness.

## 1. Introduction

Diarrhea is one of the main causes of morbidity and mortality in children under five years of age, worldwide, especially affecting low and middle-income countries (LMICs). It is estimated that diarrhea accounts for 15–30% of mortality in children of this age group, with the risk being increased by the lack of access to safe water, improved sanitation, and urgent and adequate medical care [1,2,3].

Globally, group A rotaviruses (RVA) are the main viral agents causing acute gastroenteritis (AGE) with severe dehydration in the pediatric population, with a higher incidence in children up to 24 months of age [4]. Angola, the Democratic Republic of the Congo, India, Nigeria, and Pakistan account for more than half of all RVA deaths worldwide [5]. Overall, LMICs were responsible for 80% of deaths worldwide, and Angola, with an estimated population of 33 million people in 2022 [6], has one of the highest mortality rates (5%) attributable to RVA in the world [7]. Although proper sanitation and hygiene contribute to the prevention of diarrheal disease, the ubiquitous nature of RVA infection and the young age of infected infants makes this virus particularly dangerous [8]. The Angolan population is very young, with an average age of 20.6 years. About 47.3% of the population is found in the age group of 0–14 [6]. RVA belongs to the genus Rotavirus (family Sedoreoviridae) and is a non-enveloped, wheel-shaped virus with three concentric protein layers determining a triple capsid structure [9]. The symptoms associated with RVA infection are mainly watery diarrhea and vomiting leading to excessive loss of fluids and electrolytes [10]. Besides the implementation of sanitary measures, vaccination significantly contributes to disease prevention.

In this context, two oral, live-attenuated RVA vaccines, Rotarix^®^ (Glaxo Smith Kline (GSK) Biologicals, Rixensart, Belgium) and RotaTeq^®^ (Merck Sharp and Dohme (MSD), White River, PA, USA) have been licensed for global use since 2006. Rotarix^®^ is a monovalent (RV1) vaccine derived from the human isolate G1P [8], while RotaTeq^®^ is a pentavalent (RV5) vaccine consisting of a mixture of mono-rearrangements of human and bovine RVAs that carry genes encoding for human RVA proteins G1, G2, G3, G4, and P [8] inserted into the bovine G6P [5] strain [11]. Both vaccines were considered highly effective in phase III clinical trials [12,13,14], and have been included in the childhood vaccination schedule of 123 countries since January 2023 [15].

As over 15 years have passed since their introduction in many countries, a significant number of effectiveness studies have been conducted both in LMICs and high-income countries [16]. It was shown, for example, that the Rotarix^®^ vaccine was less effective (58%) in countries with high mortality rates when compared to countries with middle (67%) or low mortality (83%) rates. However, the Rotarix^®^ vaccine significantly decreased the occurrence of diarrheal diseases and related mortality [17].

In Angola, before the introduction of the Rotarix^®^ vaccine, between 2012 and 2013, a study aimed at determining the RVA detection rate was conducted in four provinces: Huambo, Luanda, Zaire, and Cabinda. Results showed that, at the community level, the detection rate of RVA was 35% among children under 5 years of age diagnosed with AGE [18]. Since 2014, Rotarix^®^ has been administered nationwide in a two-dose schedule, as part of the governmental Extended Program for Vaccination (EPV).

Despite some progress being made towards the improvement of sanitary conditions, as well as the implementation of a nationwide RVA vaccination program, there is a significant lack of information regarding the effectiveness of these public health measures. In particular, no studies have yet been conducted to determine changes in the clinical profile, prevalence, and disease severity in children diagnosed with AGE after the introduction of the Rotarix^®^ vaccine in Angola.

Here, we performed a hospital-based study to estimate the detection rate of RVA infection, and the severity of AGE in children under five years of age, in six hospitals of Luanda Province in the post-vaccination period. Data concerning the socio-demographic profile of children, disease severity, nutritional status, access to potable drinking water, sanitation facilities, and vaccination status are presented and discussed.

## 2. Materials and Methods

### 2.1. Study Design

**Figure 1:**
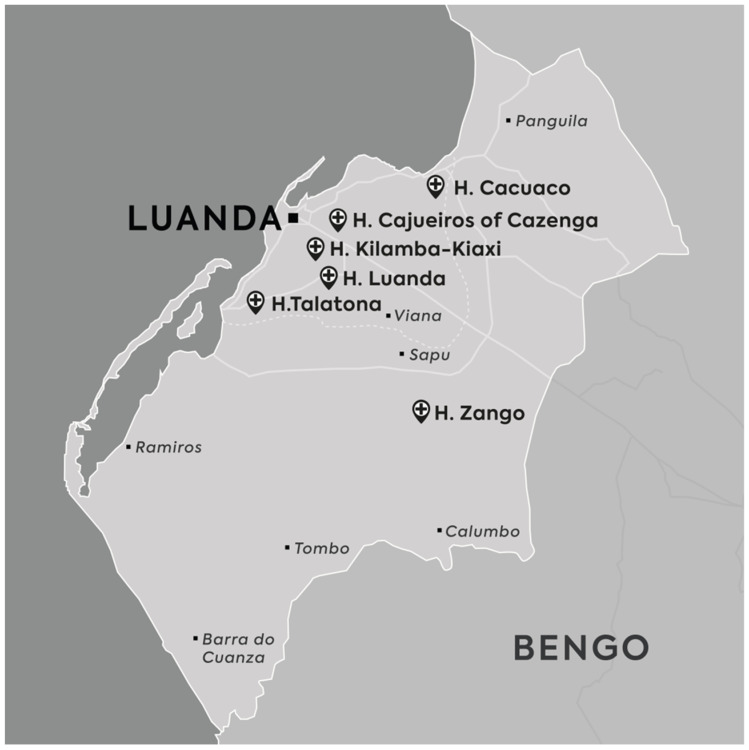
Geographical location of the study sites in the Province of Luanda, Angola. Hospitals participating in this study were pointed out by specific signs.

A cross-sectional hospital-based study was conducted from April 2021 to May 2022 at the pediatric emergency and inpatient services of 6 public hospitals from Luanda Province, namely Luanda General Hospital (H. Luanda), Geral Hospital Cajueiros of Cazenga (H. Cajueiros of Cazenga), Geral Hospital of Kilamba Kiaxi (H. Kilamba Kiaxi), Municipal Hospital of Talatona (H. Talatona), Municipal Hospital of Zango (H. Zango), and Municipal Hospital Cacuaco (H. Cacuaco) (Figure 1).

During this period, Luanda was challenged by the COVID-19 pandemic, which caused significant concern in public health services, namely those related to the supply of clean water and proper basic sanitation. The climate of this province is subtropical, characterized by the rainy and warm, as well as the cold and dry, seasons. Luanda is a highly populated city, with around 9 million inhabitants and a population density of 482/km^2^ [6].

The single-fecal samples from 1.251 children under five years of age admitted at the emergency or inpatient ward of the hospitals and previously diagnosed with AGE were used to estimate the RVA detection rate.

A case of AGE was considered when the child had diarrhea, meaning three or more fecal bowel movements and more softened stool consistency than normal during a period of 24 h, and/or vomiting [19]. Cases of children with immune deficiency, chronic diarrhea (more than 14 days of disease), nosocomial diarrhea, and prior use of antibiotic medication were excluded from the study. Socio-demographic variables including date of birth, gender, information on breastfeeding practices, source of drinking water, water treatment methods, and sanitation conditions were collected. Anthropometric data were used to determine nutritional status. Weight and height were measured according to World Health Organization (WHO) standard procedures and used to calculate anthropometric indices expressed as individual z scores using the software Anthro v. 3.2.2 (Informer Technologies, Inc., Los Angeles, CA, USA), with the determination of weight for age (WAZ, low weight), weight for height (WHZ, emaciation) and height for age (HAZ, nanism).

Malnutrition was classified as mild (−2 z score < −1), moderate (−3 z score < −2), or severe (z score < −3) [20]. Clinical files were used to complement or confirm anamnesis and a direct physical examination of the child. Symptoms associated with AGE such as diarrhea and vomiting were recorded by duration and number of episodes per day. Body temperature, child activity, and signs of dehydration were also inquired about. Depending on the severity of the symptoms, each child was referred for regular follow-up or hospitalization. Caregivers were asked to answer a questionnaire while maintaining confidentiality.

The vaccination status against RVA was checked on the child-care book or the children’s vaccination card immediately after diagnosis. Caregivers who did not remember the name of the vaccine were asked to describe the route of administration. Both the RVA and the polio vaccine are administered orally and the distinction between them was made by the amount administered—either 1 mL in a syringe (Rotarix) or two drops, respectively.

The severity of AGE was evaluated using the 20-point Vesikari Clinical Severity Scoring System Scale [21]. Data were collected through a structured and adapted epidemiological survey form tested and used in previously described studies [18,22,23].

### 2.2. Sampling and RVA Detection

The 1251 stool samples were collected in sterile containers and screened for RVA using the WHO-approved SD Bioline^TM^ Rotavirus (Abbott, Chicago, IL, USA) rapid one-step antigen immunochromatographic assay. The rapid test was performed in a BSL-2 facility by a laboratory technician trained for RVA detection using standardized operating procedures, individual protective equipment, and following all appropriate biosafety measures. The results were disclosed and recorded in the patient’s treatment plan.

The epidemiological information sheets have been previously tested to reduce the bias of measures, making sure that the questions were sensitive enough to identify the variable of interest. The research assistants were trained in specific standardized data collection procedures and the equipment used, such as a digital thermometer, measuring tape, and a scale, were inspected daily to ensure the correct flow of data.

### 2.3. Statistical Analysis

Data were analyzed using IBM SPSS software, version 25 (IBM Corps., 2011, Armonk, NY, USA). The absolute and relative frequencies of categorical variables were determined using the Chi-square test or Fisher exact test. Descriptive statistics of the variables were generated by comparing the means ± standard deviation or medians ± interquartile amplitude, depending on whether the distribution was normal or not. The T-student or Mann–Whitney tests were used in the same way in the comparison of distributions. The study employed binary logistic regression to assess the significance of the prevalence of RVA and AGE with a common risk factor, using an adjusted OR, 95% confidence interval, and a significance level *p* ≤ 0.05 (α = 5%).

### 2.4. Ethical Considerations

This study was approved by the Independent Ethics Committee of the Faculty of Medicine, Agostinho Neto University, Luanda, Angola (Reference n° 12/2021). Before enrolling each child, informed and voluntary consent was obtained from parents or legal guardians.

## 3. Results

### 3.1. RVA Detection and Socio-Demographic Profile of the Children Enrolled in the Study

This study included 1251 children, of which 53.1% were girls and 46.9% were boys. The total detection rate of RVA was 57.8% (723/1251), representing 55.2% of girls (399/723) and 44.8% of boys (324/723) (Table 1).

The majority of these children, 78% (564/723), belong to the age group of 1–6 months (Table 2).

No significant association was found between RVA infection and gender (*p* = 0.90), but the mean age was significantly lower in the group that tested positive (13 ± 12 vs. 14 ± 15, *p* = 0.02). In addition, a positive association between RVA-positive infection and younger age (1–6 months) was found (*p* < 0.001) (Table 2). The detection rate of RVA in each hospital included in this study showed that H. Kilamba Kiaxi and H. Luanda had the highest prevalence of69.4% (152/582) and 61.3% (357/582), respectively. H. Cajueiros of Cazenga has a prevalence of 56.4% (61/108), H. Zango of 55.8% (63/113), and H. Cacuaco with 47.7% (53/111) showed intermediate prevalences, while the lowest rate was detected in H. Talatona with 31.3% (37/118) (Table 1).

### 3.2. RVA Prevalence and Severity of AGE

In Angola, in the routine vaccination program, children are eligible to receive the first dose of Rotarix at two months of age and the second dose is administered at four months of age, according to the recommendation of the WHO. Data about the distribution of RVA detection rate and severity of AGE (mild, moderate, and severe) are depicted in Table 3.

We found the highest prevalence of RVA-positive cases in the youngest age group of 1–6 months (60.3%). Furthermore, this age group had the highest frequency of severe cases of AGE (81.0%; *p* < 0.001).

The assessment of nutritional status allowed us to determine that the prevalence of score > −2SD vs. ≤ −2SD showed a tendency to be higher when considering weight-for-age parameters (61.2% vs. 56.7%), followed by weight for height (61.5% vs. 56.3%) and height for age (57.9 vs. 57.4%). However, these results did not carry statistical significance (*p* > 0.05). In addition, the severity of the AGE was more pronounced in groups > −2SD with the only exception being the height for the age group (79.7% vs. 80.3%).

RVA-positive cases of infection were also more frequent (68.1%) and more severe (92.6%) in children with lethargy (*p* < 0.001).

Interestingly, we found a positive association between exclusive breastfeeding, the prevalence of RVA (66.1%), and the high severity of AGE (86.4%) (*p* < 0.001); however, all children who were never breastfed had severe AGE.

Sanitary conditions, including access to potable water, were also positively associated with AGE severity. We observed a high detection rate of RVA in individuals who used undisclosed water sources (60.8%), consumed treated water (59.8%) or bleach-treated water (60.1%), and used latrines with running water (61.0%). However, the association of these conditions with RVA prevalence was not found to be statistically significant. In contrast, the severity of AGE seems to be positively associated with the use of private latrines with running water, drinking water from public fountains (88.9%), or untreated water (80.4%). Children who used no latrines or shared latrines with running water were also at higher risk of contracting more severe disease (100%) (*p* < 0.001). Strikingly, vaccination did not seem to protect children against RVA infection nor the development of severe symptoms of the disease. In fact, our data show that children who received no doses of Rotarix were at a lower risk of infection (*p* = 0.05) and did not develop more severe symptoms when compared to children who received one or two doses of the vaccine (*p* = 0.60).

### 3.3. Clinical Severity of RVA Infection

According to Vesikari’s Clinical Severity Score, 644 (89.0%) of the 723 RVA-positive cases had severe symptoms, 72 cases (10.0%) were classified as moderate, and only 7 (1.0%) were considered mild. Among the different signs and symptoms of the clinical severity of the disease, diarrhea duration for 1 to 4 days (68.9%) and 6 or more episodes of diarrhea per day (69.7%) were the most common. Most children (76,9%) reported vomiting lasting longer than 3 days (76.9%) and more than 5 episodes per day (82.7%). Fever was not confirmed in only 4 children (0.6%), while, during hospital consultation, 498 febrile children (68.9%) had temperatures between 37.1 and 38.4 °C.

### 3.4. Factors Associated with Infection and Severity of Disease

The statistical significance of the association of children’s characteristics with RVA infection and the severity of AGE was also evaluated using a logistic regression analysis. However, no association could be found with most variables (Table 4).

The probability of infection from weaned children and children receiving complementary breastfeeding increased when compared to those breastfeeding: (OR = 3.38 [1.85–6.20]) and (OR = 2.17 [1.25–3.75]), respectively. Moreover, the use of a shared latrine without running water also increased the risk of infection (OR = 2.06 [1.24–3.42] and severity of AGE (OR = 1.99 [1.35] –2.96]) by approximately 2 times when compared to home running water users, as shown in Table 4.

### 3.5. Seasonality of RVA Infection

**Figure 2:**
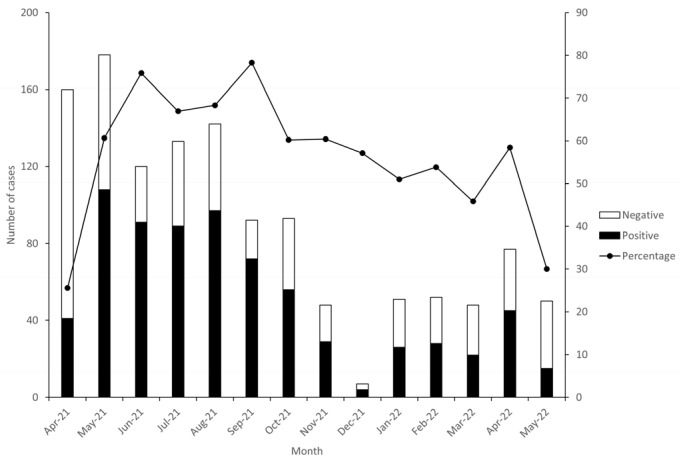
Seasonal variation in RVA prevalence in six hospitals of Luanda Province from April 2021 to May 2022.

The seasonality of RVA infection during the study period, from April 2021 to May 2022, among AGE cases treated at Luanda Province hospitals is depicted in Figure 2.

At the beginning of the dry season (May to September 2021), from the peak in May 2021 (108/177, 60%) and with monthly fluctuations, the RVA detection rate decreased, reaching a minimum point in December 2021 (4/7, 57%). In the rainy season (October 2021 to May 2022), the downward trend was maintained with monthly fluctuations. This agrees with observations made in other African studies [24].

## 4. Discussion

The present hospital-based study estimated the prevalence of RVA infection in children under 5 years of age hospitalized with AGE in six reference hospitals of Luanda Province. In these settings, we found a high percentage of infected children (57.8%), indicating RVA as an important cause of diarrhea. RVA detection rate was higher than that previously reported by Esteves et al. in 2016 (35%) [18], or that observed by Gasparinho et al. in 2017 (25.1%) [22] during community-based studies. These studies were conducted before the introduction of Rotarix in the EPV. Additionally, we also investigated several socio-demographic characteristics of the studied population in order to obtain relevant epidemiological data on RVA infection in the target population after the introduction of the vaccine in Luanda Province. The groups of children most significantly affected by RVA were found to be the youngest, at 0–6 and 7–12 months of age. These results indicated that the prevalence of RVA decreased with increasing age, supporting a tendency already reported in other studies [25].

It is hypothesized that the reason for the increased disease susceptibility observed in the age group of 1 to 6 months may be due to an eventual high exposure of children to contaminated objects. In fact, it is known that children belonging to this age group are in the early stages of development and exhibit behavioral patterns that are prone to potentiate an increased exposure to unsanitized objects. In addition to this, natural immunity has also been suggested as one of the factors influencing the decrease in the incidence of RVA with age [26].

Although this study did not show statistically significant differences in the burden of RVA disease in relation to gender, positive cases of infection were more frequently found in girls than in boys (60.1% vs. 55.2%, respectively). This observation supports the results of a previous study performed in Zambia [27] but is in contrast with other earlier reports where the RVA detection rate was found to be higher in boys [2,18,22].

High RVA infection rates, as well as the low efficacy of vaccination in LMICs, have been hypothesized to be influenced by several factors, including malnutrition [28]. Thus, we also investigated the nutritional status of the children who participated in this study. A previous study carried out at the community level in Northern Angola before the introduction of Rotarix in the EVP reported a slightly lower prevalence of RVA infection (38%) in underweight children from both rural and urban areas, when compared to the prevalence found by us (43.3%) [22]. Interestingly, in this study, we found a positive correlation between underweight children and severity of disease (*p* = 0.04). This observation seems to support the view that malnutrition per se is not a factor influencing the RVA infection rate, but rather its severity, probably due to an eventual partial impairment of the immune status of children.

A study carried out in Mozambique that aimed to determine the frequency and potential risk factors associated with RVA infection reported a prevalence of the virus of 42.7% in malnourished children, a result similar to the one found in this study [29]. In other studies conducted in Africa, weight loss was associated with both more severe forms of diarrhea and a higher prevalence of RVA infection. The impact of the nutritional status on susceptibility to RVA infection remains controversial and far from being completely understood [22,28]. In the present study, signs of malnutrition were quite evident in a high proportion of children who participated in the study. Although underweight and emaciated children appeared more prone to infection with RVA, no statistically significant association could be established.

Early weaning and the introduction of complementary feeding are known to have significant implications for children’s health since the immune system is still immature, a status that may affect bowel function, leading to an increased risk of diarrheal diseases and stunted growth [30]. In this study, we found that a high proportion of RVA-infected children (96.7%) were fed either complementary or exclusively with breast milk. In Angola, the national guidelines on breastfeeding practices comply with the WHO guidelines, which recommend exclusive breastfeeding up to 6 months of age and continued breastfeeding until 2 years of age with appropriate complementary feeding. However, the specific role of breastfeeding in the prevention of RVA diarrhea is still not well established, although it is generally considered to contribute to at least reducing the severity of the disease [31].

A high burden of diarrheal disease was previously associated with limited access to safe water and poor sanitation, factors that facilitate the transmission of enteric pathogens and are of special relevance in LMICs [32]. In a 2012 study in sub-Saharan Africa from the Global Enteric Multicenter Study (GEMS), 83% of urban populations and 49% of rural populations had access to improved water sources. However, access to sanitation facilities is much lower both in urban populations (43%) and rural populations (23%) [33]. Improving these conditions has become a strong public health priority and it is one of the WHO Millennium Development Goals to be continued within the scope of the 2030 Sustainable Development Agenda.

In the present study, 57.9% of the parents or legal guardians with a child who has tested positive for RVA declared using water from a tank and that the most common domestic treatment method included the use of bleach. However, for home drinking water treatment procedures, it is very difficult to ascertain whether the correct concentration of bleach is being used. Consequently, the observations herein reported must be taken with great caution. It is noteworthy that 55.8% of RVA-positive children were mentioned as ingesting untreated water. The importance of adequate and regular implementation of the domestic treatment of drinking water and its adequate storage needs to be stressed regarding its potential impact on the transmission of RVA and other enteric pathogens.

This study showed a significant association of RVA infection with hygiene practices related to the household’s sanitary facilities, and the use of a shared latrine without running water (OR = 2.06 [1.24–3.42] when compared to running water in domestic users (OR = 1.99 [1.35–2.96]). Strikingly, the RVA detection rate was higher when the use of private latrines with running water was reported (61.0%). Furthermore, the RVA detection rate was lower in users of shared latrines without running water (37.5%). Disinfection, implementation of hygienic measures, as well as adequate and healthy sanitation behaviors, should be promoted and implemented at the household level, particularly in improved sanitation facilities using domestic latrines.

Concerning clinical features and symptoms potentially associated with RVA infection, those presented by these patients showed a strong statistical significance when compared to control groups comprising individuals with the same symptoms that were diagnosed with AGE not caused by RVA. Most RVA-positive children had more than six episodes of diarrhea in less than 24 h. Vomiting, fever, and dehydration were observed more frequently in children with AGE caused by RVA than in those not infected with RVA, which agrees with results from a previous study from our group [23,33]. A study in Zaria, Nigeria, found that 78.4% of children were dehydrated due to RVA infection [2]. This association has also been previously demonstrated in other sub-Saharan African countries [34]. Vomiting is a common symptom in children infected with RVA, and its clinical management requires special attention. In fact, prolonged vomiting in these children can quickly deplete the body of water, leading to dehydration, and profoundly affecting the electrolyte balance.

The severity of AGE assessed by the 20-point Vesikari Clinical Severity Scoring System used in this study showed a distribution of severe diarrhea similar among children who were positive for RVA when compared to those who were RVA-negative. Comparing the severity of RVA diarrhea reported in different studies is a difficult task, mainly due to the use of various forms of classification systems. This can be illustrated by some studies that have described the severity of RVA-associated diarrhea using the Vesikari Clinical Scoring System, and the need for hospitalization as a mark of disease severity [35], while others chose to assess diarrhea severity using hydration status.

Although several studies from sub-Saharan African countries point to a higher prevalence of RVA infection in the dry season [36], no seasonality of RVA infection was evident in our year-round study. However, it seems plausible that there is an apparent increase in diarrhea cases admitted to Luanda hospitals during the dry season, showing the possibility that other enteric agents may be involved as etiological agents. These findings correlate well with data reported previously by Coulson et al. (2018) [37]. We can speculate that this may be due to an improved potential “survival” of RVA particles in the colder months. In addition, a hypothesis has been raised proposing that the spread of RVA in the cold season happens in relatively low humidity conditions during the winter months, combined with soil drying. In the broader African geographical context, the seasonality of RVA infections varied throughout Africa, reflecting the differences in climate conditions.

The vaccination status against RVA was verified considering the records in vaccination cards. Regarding RVA vaccine coverage, Angola’s National Directorate of Public Health reported an RVA vaccination coverage in Luanda of 74% for the first dose and 62% for the second dose in 2021, a picture like that found in Ethiopia (95% CI: 66.8, 72.5) [38].

Our study showed that vaccine coverage does not seem to be sufficient, neither for the first nor for the second doses, as a high prevalence of RVA was observed even among children vaccinated with two doses of Rotarix (57.6%). Furthermore, our study suggests that appropriate cold chains, storage, and administration conditions of vaccines must be tightly monitored. The improvement of sanitary conditions and implementation of programs aimed at improving habits of hygiene and health literacy are priorities to reduce the burden of RVA.

This is a cross-sectional study, which does not allow measuring the causality between the variables and RVA infection. Further research should be conducted on RVA molecular epidemiology and surveillance considering the possibility of including community-based studies with more municipalities, districts, and communities to better understand the real situation of RVA infection and the severity of the disease and try to answer the reasons for the low effectiveness of vaccination in the surveyed cohort of children.

### Limitations of the Study

This study is hospital-based. Accordingly, results cannot be extrapolated to the entire population and may reflect mainly the most severe cases of children with diarrhea who sought out health services. Sample size and scope may also be considered a limitation since only six hospitals were engaged and, therefore, it is not possible to consider it as a provincial-wide study. This study being cross-sectional does not allow us to establish cause-and-effect relationships between pairs of variables. Assessment of incidence values is also not feasible using a study design based on a cross-sectional approach.

This study was conducted in Luanda during the COVID-19 pandemic, when waste management and sanitary measures were limited given the heavy restrictions imposed by health authorities. It is possible that a general deterioration of public services, particularly those related to health care and the maintenance of sanitation procedures and standards, contributed to the worsening of the epidemiological picture observed in this study.

## Figures and Tables

**Table 1:** Characteristics of the study samples.

Variables		Total n (%)	RV (+) n (%)	RV (−) n (%)	*p*-Value
**Number of Samples**		**1251 (100)**	**723 (57.8)**	**528 (42.2)**	--
**Gender**	Male	587 (46.9)	324 (44.8)	263 (50.0)	0.080 *
	Female	664 (53.1)	399 (55.2)	265 (50.0)	
**Median age ± IQR**	--	--	13 ± 12	14 ± 15	**0.027** **
**Hospitals in Luanda Province**	Luanda	582 (46.5)	357 (613)	225 (38.6)	<**0.001** *
	Kilamba Kiaxi	219 (17.5)	152 (69.4)	67 (30.6)	
	Zango	113 (9.0)	63 (55.8)	50 (44.2)	
	Cacuaco	111 (8.9)	53 (47.7)	58 (52.2)	
	Cajueiros	108 (8.6)	61 (56.4)	47 (43.5)	
	Talatona	118 (9.4)	37 (31.3)	81 (68.6)	

* Chi-square test; ** Mann–Whitney test.

**Table 2:** Socio-demographic profile of children with AGE enrolled in this study.

	Rotavirus Infection			Severity of Acute Gastroenteritis			
Variables	Positive n (%)	Negative n (%)	*p*-Value	Mild n (%)	Moderate n (%)	Severe n (%)	*p*-Value
**Number of Samples** **n = 1251**	723 (57.8)	528 (42.2)	--	46 (3.7)	207 (16.5)	998 (79.8)	--
**Age group (months)**							
1–6 (n = 935)	564 (60.3)	371 (39.7)	**<0.001**	20 (2.4)	158 (16.9)	757 (81.0)	**<0.001**
7–12 (n = 267)	148 (55.4)	119 (44.6)		21 (7.9)	41 (15.4)	205 (76.8)	
13–24 (n = 35)	7 (20.0)	28 (80.0)		4 (11.4)	4 (11.4)	27 (77.1)	
24–59 (n = 14)	4 (28.6)	10 (71.4)		1 (7.1)	4 (28.6)	9 (64.3)	
**Nutritional status**							
Underweight (Weight-for-age z score)							
WAZ ≤ −2 SD (n = 944)	535 (56.7)	409 (43.3)	0.15	42 (4.4)	162 (17.2)	740 (78.4)	**0.01**
WAZ > −2 SD (n = 307)	188 (61.2)	119 (38.8)		6 (2.0)	43 (14.0)	258 (84.0)	
Wasting (Weight-for-height z score)							
WHZ ≤ −2 SD (n = 898)	506 (56.3)	392 (43.7)	0.09	39 (4.3)	154 (17.1)	705 (78.5)	0.06
WHZ > −2 SD (n = 353)	217 (61.5)	136 (38.5)		07 (2.0)	53 (15.0)	293 (83.0)	
Stunting (Height-for-age z score)							
HAZ ≤ −2 SD (n = 183)	105 (57.4)	78 (42.6)	0.90	07 (3.8)	29 (15.8)	147 (80.3)	0.69
HAZ > −2 SD (n = 1068)	618 (57.9)	450 (42.1)		39 (3.7)	178 (16.7)	851 (79.7)	
**Lethargy**							
No (n = 612)	288 (47.1)	324 (52.9)	**<0.001**	46 (7.5)	160 (26.1)	406 (66.3)	**<0.001**
Yes (n = 639)	435 (68.1)	204 (31.9)		0 (0.0)	47 (7.4)	592 (92.6)	
**Breastfeeding**							
Exclusive (n = 257)	170 (66.1)	87 (33.9)	**<0.001**	2 (0.8)	33 (12.8)	222 (86.4)	**<0.001**
Complementary (n = 918)	527 (57.4)	391 (42.6)		34 (3.7)	162 (17.6)	722 (78.6)	
Weaned (n = 72)	25 (34.7)	47 (65.3)		10 (13.9)	12 (16.7)	50 (69.4)	
Never (n = 4)	1 (25.0)	3 (75.0)		0 (0.0)	0 (0.0)	4 (100)	
**Water source**							
Public fountain (n = 180)	98 (54.4)	82 (45.6)	0.71	3 (1.7)	17 (9.4)	160 (88.9)	**0.004**
Home running water (n = 176)	105 (59.7)	71 (40.3)		9 (5.1)	30 (17.0)	137 (77.8)	
Water tank (n = 821)	475 (57.9)	346 (42.1)		33 (4.0)	139 (16.9)	649 (79.0)	
Other source (n =74)	45 (60.8)	29 (39.2)		1 (1.4)	21 (28.4)	52 (70.3)	
**Drinking water treatment**							
Yes (n = 622)	372 (59.8)	250 (40.2)	0.15	16 (2.6)	114 (18.3)	492 (79.1)	**0.03**
No (n = 629)	351 (55.8)	278 (44.2)		30 (4.8)	93 (14.8)	506 (80.4)	
**Water treatment method**							
Bleach (n = 612)	368 (60.1)	244 (39.9)	0.15	16 (2.6)	112 (18.3)	484 (79.1)	0.15
Boiled water (n = 10)	4 (40.0)	6 (60.0)		0 (0.0)	2 (20.0)	8 (80.0)	
None (n = 629)	351 (55.8)	278 (44.2)		30 (4.8)	93 (14.8)	506 (80.4)	
**Sanitation facilities**							
Without latrine (n = 6)	1 (16.7)	5 (83.3)	**<0.001**	0 (0.0)	0 (0.0)	6 (100.0)	**<0.001**
Private with running water (n = 1048)	639 (61.0)	409 (39.0)		33 (3.1)	157 (15.0)	858 (81.9)	
Private without running water (n = 38)	16 (42.1)	22 (57.9)		3 (7.9)	8 (21.1)	27 (71.1)	
Shared with running water (n = 143)	61 (42.7)	82 (57.3)		8 (5.6)	35 (24.5)	100 (69.9)	
Shared without running water (n = 16)	6 (37.5)	10 (62.5)		2 (12.5)	7 (43.8)	7 (43.8)	
**Number of vaccine doses**							
None (n = 85)	40 (47.1)	45 (52.9)	0.05	4 (4.7)	13 (15.3)	68 (80.0)	0.55
One (n = 275)	170 (61.8)	105 (38.2)		10 (3.6)	54 (19,6)	211 (76.7)	
Two (n = 891)	513 (57.6)	378 (42.4)		32 (3.6)	140 (15.7)	719 (80.7)	

**Table 3:** Distribution of RVA-positive cases (n = 723) considering the Vesikari scoring for AGE.

Parameters		n (%)	Points
**Max. n° of diarrheal stools per 24 h period during the course of disease**	1–3	68 (9.4)	**1**
	4–5	151 (20.9)	**2**
	≥6	504 (69.7)	**3**
**Diarrhea duration (days)**	1–4	498 (68.9)	**1**
	5	110 (15.2)	**2**
	≥6	115 (15.9)	**3**
**Max. nº of vomiting episodes per 24 h period during the course of disease**	0	118 (16.3)	**0**
	1	0 (0.0)	**1**
	2–4	7 (1.0)	**2**
	≥5	598 (82.7)	**3**
**Vomiting duration (days)**	0	118 (16.3)	**0**
	1	4 (0.6)	**1**
	2	45 (6.2)	**2**
	≥3	556 (76.9)	**3**
**Temperature (°C)**	≤37	4 (0.6)	**0**
	37.1–38.4	498 (68.9)	**1**
	38.5–38.9	113 (15.6)	**2**
	≥39.0	108 (14.9)	**3**
**Dehydration status**	None	5 (0.7)	**1**
	Mild dehydration	296 (40.9)	**2**
	Severe dehydration	422 (58.4)	**3**
**Treatment**	Rehydrated	85 (11.8)	**1**
	Admitted to hospital	638 (88.2)	**2**

**Table 4:** Logistic regression analysis of children characteristics, RVA infection, and AGE.

Children’s Characteristics	Rotavirus Infection			Severe Acute Gastroenteritis		
	Β	Odds Ratio [95% CI]	*p*-Value	Β	Odds Ratio [95% CI]	*p*-Value
**Female** (ref: Male)	0.187	1.21 (0.94–1.55)	0.14	0.015	1.02 (0.75–1.38)	0.92
**Child age** (ref: 24–59 months)						
1–6	0.973	2.65 (0.78–8.95)	0.11	0.359	1.43 (0.45–4.58)	0.54
7–12	1.017	2.77 (0.79–9.62)	0.11	0.430	1.54 (0.46–5.12)	0.48
13–24	0.088	1.09 (0.26–4.61)	0.90	0.572	1.77 (0.43–7.26)	0.42
**Breastfeeding** (ref.: Exclusive)						
Weaned	1.218	3.38 (1.85–6.20)	**<0.001**	0.894	2.45 (1.23–4.85)	**0.01**
Complementary	0.773	2.17 (1.25–3.75)	**0.006**	0.377	1.46 (0.81–2.62)	0.20
**Nutritional status**						
Weight-for-height z score (ref.: WHZ > −2SD)	0.142	1.15 (0.66–2.01)	0.61	−0.250	0.78 (0.41–1.49)	0.44
Height-for-age z score (ref.: HAZ > −2SD)	−0.073	0.93 (0.63–1.34)	0.69	−0.130	0.88 (0.56–1.38)	0.05
Weight-for-age z score (ref.: WAZ > −2SD)	0.042	1.04 (0.58–1.88)	0.88	0.632	1.88 (0.94–3.78)	0.07
**Drinking water source** (ref.: Home running water)						
Public fountain	0.274	1.32 (0.92–1.90)	0.14	–0.030	0.98 (0.64–1.49)	0.89
Water tank	0.082	1.09 (0.64–1.84)	0.76	–0.410	0.67 (0.38–1.18)	0.16
Other source	−0.155	0.86 (0.61–1.21)	0.37	0.722	2.06 (1.24–3.42)	**0.005**
**Water treatment** (ref: Yes)	0.416	1.52 (0.39–5.91)	0.54	–0.440	0.65 (0.13–3.28)	0.59
**Water treatment method** (ref.: Boiled)						
Bleach	0.843	2.23 (0.60–9.02)	0.22	–0.160	0.85 (0.19–4.30)	0.84
**Sanitation facilities** (ref: Private with running water)						
Shared without running water	0.768	2.06 (1.24–3.042)	**<0.001**	0.691	1.99 (1.35–2.96)	**0.001**
Shared with running water	−0.060	0.94 (0.43–1.97)	0.87	0.029	1.03 (0.47–2.29)	0.94

## Data Availability

Dataset available on request from the authors.
